# Intra-procedural CT control versus ultrasound only in ultrasound-guided thermal ablation of colorectal liver metastases: a single-centre cohort study

**DOI:** 10.1007/s00464-026-12912-4

**Published:** 2026-05-22

**Authors:** Trygve Syversveen, Ingrid Schrøder Hansen, Knut Brabrand, Kristoffer Watten Brudvik, Ida Björk, Daniel Østergaard, Olaug Villanger, Bård Ingvald Røsok, Kristoffer Lassen, Bjørn von Gohren Edwin, Sheraz Yaqub, Åsmund Avdem Fretland

**Affiliations:** 1 Department of Radiology, Oslo University Hospital, Oslo, Norway; 2 Department of Hepato-Pancreato-Biliary Surgery, Oslo University Hospital, Oslo, Norway; 3 The Intervention Centre, Oslo University Hospital, Oslo, Norway; 4 Institute of Clinical Medicine, University of Oslo, Oslo, Norway; 5 Department of Clinical Medicine, University of Tromsø, Tromsø, Norway

**Keywords:** Ablation margins, Colorectal liver metastasis, CT control, Local tumour progression, Thermal ablation, Ultrasound guiding

## Abstract

**Background:**

Local ablative treatment is increasingly used for small colorectal liver metastasis (CRLM). Adequate ablative margins are essential for achieving local control, yet reported rates of local tumour progression (LTP) vary widely. This study compared LTP rate after ultrasound (US)-guided ablation of CRLM performed with versus without intraoperative CT control.

**Methods:**

All percutaneous US-guided ablations for CRLM performed in a single centre from 2018 to 2021 were reviewed for LTP. Procedures with intraprocedural CT (CT group), allowing immediate re-ablation for margin extension, were compared with procedures without intraprocedural CT control (OR group).

**Results:**

A total of 152 procedures in 137 patients were analysed. In the CT group, 5 LTP events (8.0%) occurred after 62 procedures, affecting 5 of 111 CRLM (4.5%). In the OR group, 20 LTP events (22.2%) occurred after 90 procedures, affecting 22 of 132 CRLM (16.6%) (procedure-based and lesion-based *p* = 0.0035 and *p* = 0.0255), respectively. For CRLM ≤ 10 mm visualized with US, LTP occurred in 1/50 lesions (2%) in the CT group and 0/51 lesions (0%) in the OR group (*p* = 0.495). Most LTP occurred with synchronous recurrence elsewhere in the liver (CT group 3/5 [60%], OR group 16/22 [73%]). Only minor complications, Accordion grades 1–2, were observed with similar frequency (8% vs. 12% in CT group and OR group, respectively).

**Conclusion:**

Intraprocedural CT control was associated with substantially lower LTP, suggesting that CT control should be considered standard when available. US-only ablation may remain appropriate for small, clearly visualized lesions.

**Graphical abstract:**

**Figure:**
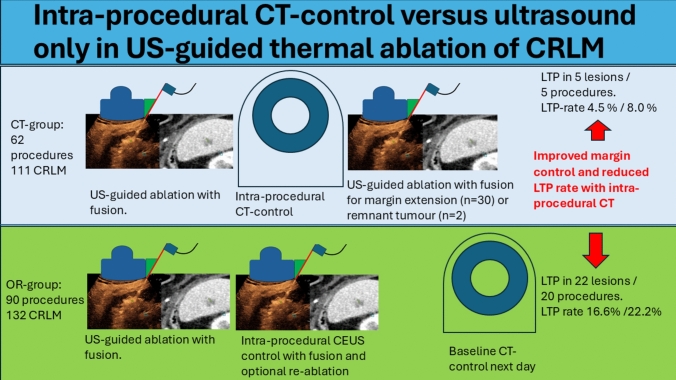

Local ablative treatment has been used with increasing frequency in the treatment of colorectal liver metastasis (CRLM). For resectable CRLM, resection is still the preferred treatment [1], but thermal ablation has gained importance supported by recent randomized and prospective trials showing comparable survival to resection and lower morbidity [2, 3].

Achieving complete tumour coverage with an adequate ablative margin remains critical. Local tumour progression (LTP) rates after ablation vary widely from 12 to 54% in large series [4, 5], reflecting differences in imaging guidance, tumour biology, and margin assessment. In computed tomography (CT)-guided liver tumour ablation, the use of software-assisted margin confirmation improved the minimal ablative margin compared to visual assessment [6]. Many centres, however, rely on ultrasound (US) guidance alone as it is real-time, inexpensive, and widely available. Although low LTP rates have also been reported with US-guided thermal ablation both with a percutaneous and an open approach [7–9], US has limitations in confirming margins.

With an increased use of thermal ablation, a low-cost alternative with US-guided thermal ablation and control could still be of interest, provided that sufficient local tumour control could be achieved. Whether intraprocedural CT control confers a meaningful reduction in LTP when US remains the primary targeting modality is unclear. Clarifying the added value of CT control is important for optimizing resource allocation and determining which lesions may still be adequately managed with US-only.

The aim of this study was to compare LTP rates after US-guided thermal ablation with or without intraprocedural CT control. We also evaluated whether tumour size modified the effect of CT control and reported the management of LTP to assess its clinical consequences.

## Material and methods

### Study design and ethical approval

This cohort study included consecutive patients undergoing percutaneous thermal ablation for CRLM from January 2018 to December 2021 at a single tertiary hepatobiliary centre. The study was approved by the Data Protection Officer, Oslo University Hospital (Register 21/04438, case 25/25703).

### Patient selection

**Fig. 1:**
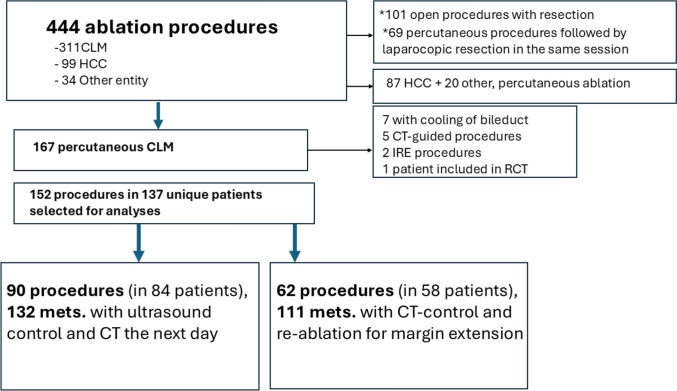
Flowchart of patient and procedure selection. Five patients were treated in both the OR and the hybrid CT lab during the inclusion period. All procedures were included in lesion-level and procedure-level LTP analyses

All patients were evaluated in multidisciplinary tumour board (MDT) meetings before treatment. Patients were eligible if they underwent US-guided percutaneous thermal ablation during the study period. The study interval encompassed the institutional transition from next-day CT control to intraoperative CT availability. From March 2019, a hybrid OR/CT lab was available, and inclusion ended at the start of inclusion to the New Comet study [10]. A flow chart for the selection is given in Fig. 1. During this period, most thermal ablations were performed as part of a parenchymal-sparing strategy, i.e. ablation was chosen if the expected parenchymal loss would be significantly less with ablation than with resection, typically for deeply located or small tumours. Other indications for ablation were frail patients or cases where resection was considered technically challenging.

Procedures were assigned to two groups. In the CT group thermal ablation was performed in a hybrid OR/CT lab with the possibility of immediate re-ablation. In the OR group, patients were treated in an ordinary operating room with next-day CT margin control.

The treatment allocation was not completely random. Cases considered difficult due to tumour localization or visibility were preferably allocated to hybrid OR/CT lab, but due to limited CT availability, this could not always be achieved.

All ablations were performed with curative intent, and patients were not accepted for treatment unless all CRLM could be treated.

### Ultrasound-guided ablation procedure

All thermal ablations were performed under general anaesthesia using US (LOGIQ E10; GE Healthcare, Milwaukee, WI, USA) with a Verza™ guidance system (CIVCO, IA 52247 USA) mounted on a C1-6VN transducer (GE). Single-lung ventilation was frequently used for right-sided lesions to minimize liver movement and improve targeting stability. Image fusion with the most recent CT or magnetic resonance imaging (MRI) examination available was routinely used to enhance lesion localization. Contrast-enhanced ultrasound (CEUS) with Sonazoid (GE Healthcare) for improved visualization when targeting the tumours was also used, except for the cases where the tumours were better visualized with US without contrast. For lesions not visualized by US/CEUS, a fusion-based targeting was performed, i.e. the applicator was inserted US-guided into the localization corresponding to the localization of the CRLM on the CT/MRI used for fusion. In all cases, a CEUS control (SonoVue (Bracco Imaging Italy) or Sonazoid) with fusion was performed to assess the ablation zone after treatment. The intended ablation margin was a minimum of 5 mm, ideally 10 mm, and re-ablation performed if indicated. Thermal ablation was performed using radiofrequency ablation (RFA), Covidien Cool-Tip (Covidien Ilc, Mansfield, MA 02048 USA) or microwave ablation (MWA), and Covidien Emprint™ (Covidien Ilc, Mansfield, MA 02048 USA) (maximum 100 W effect) at the discretion of the radiologist, following manufacturer’s protocol.

### Intraprocedural CT control (CT group)

Following US/CEUS assessment, portal venous phase CT was obtained using a sliding-gantry system (Siemens SOMATOM Definition Edge, Siemens Healthineers AG). In case of visible remnant tumour or if margins were inadequate, immediate re-ablation was performed using CT–US fusion for targeting, using the control CT for fusion. Margin evaluation was based on visual assessment, and software-assisted margin confirmation was not used. A final confirmation CT after re-ablation was obtained in selected cases.

### No intraprocedural CT control (OR group)

Procedures relied solely on US/CEUS for intraprocedural ablation assessment. A contrast-enhanced CT performed the following day served as the baseline reference imaging for follow-up but did not allow re-ablation. In this setting, coverage of tumour was regarded as an acceptable result and insufficient margins were tolerated as immediate re-ablation for margin extension would imply an additional procedure.

### Imaging before and follow-up including image control after ablation

All patients had CRLM diagnosed with CT-abdomen and routinely had MRI with liver-specific contrast if not contraindicated. Tumour size was measured retrospectively on intraprocedural US/CEUS images. For CRLM not visualized with US/CEUS, the size from the most recent CT or MRI was used.

Post-procedural surveillance included contrast-enhanced CT of the chest and abdomen every 6 months. As our institution is the only centre providing liver surgery and ablation in the health region, patients with treatable recurrence were routinely re-referred, ensuring near-complete follow-up. Regarding deaths, complete follow-up was available except for one patient who moved abroad 28 months after the ablation procedure.

All available CT, MRI, and PET images were reviewed by a dedicated abdominal radiologist (TS) for the assessment of LTP, as defined by Goldberg et al. [11] and for recurrence in the liver not representing an LTP. Difficult cases were reviewed by another radiologist (DØ) and consensus was reached. The size of the treated CRLM where LTP later occurred was identified, as well as if fusion-based applicator placement in US non-visualized tumour had been performed.

### Statistical analyses

Descriptive data are summarized as the number and percentage, median with ranges or mean ± standard deviation. Continuous variables were compared using Student’s *t*-test or Mann–Whitney *U*-test; categorical variables were compared using the chi-squared test or Fisher’s exact test. Two-sided *p* < 0.05 was considered statistically significant. Survival and LTP-free survival were analysed using the Kaplan–Meier method and the log-rank test, with follow-up and survival time and LTP-free survival calculated from the date of the first ablation procedure during the study period. Patients alive at last follow-up were censored. In addition, multivariate logistic regression analysis with LTP as the dependent variable was performed to identify independent predictors for LTP. Statistical analyses were performed using SPSS 30.0 (SPSS Inc, Chicago, IL, USA).

## Results

A total of 152 percutaneous ablation procedures for CRLM were performed in 137 unique patients during the study period (Fig. 1). Of these, 90 procedures treating 132 lesions were performed in the operating room (OR group), and 62 procedures treating 111 lesions were performed in the hybrid OR/CT lab (CT group).

Baseline demographics, tumour characteristics, and procedural details are presented in Table 1. The two groups were broadly comparable, with no significant differences in age, sex distribution, prior liver interventions, lesion size categories, ablation modality (RFA/MWA), follow-up duration, or median number of metastases per patient.

**Table 1:** Main demographic and tumour characteristics at procedure time

	OR	CT	*p*-value
Gender	35W/55 M (39/61%)	30W/32 M (48/52%)	0.244
Age at procedure time	63	65	0.307
Number of patients	84	58	-
Number of procedures	90	62	-
Number of CLM treated	132	111	-
First time liver intervention procedure	51/90 (57%)	40/62 (65%)	0.332
Previous liver intervention	39/90 (43%)	22/62 (35%)	0.332
Left-sided primary cancer (per procedure)	73/90 (81%)	55/62 (89%)	0.207
Largest CLM, median/mean(range)	15/15,7 mm (5–32 mm)	13,5/15,4 mm (3–36 mm)	0.749
All CLM, mean (SD)	13.5 mm (0.60)	11.8 mm (0.70)	0.075
CLM > 30 mm	1 (0.8%)	2 (1,8%)	0.654
CLM 21–30 mm	21 (15.9%)	11 (9.9%)	0.168
CLM 11–20 mm	54 (40.9%)	42 (37.8%)	0.731
CLM ≤ 10 mm	51 (38.6%)	50 (45.0%)	0.312
CLM US-non-visualized, fusion-based targeting	5 (3.8%)	6 (5.4%)	0.350
Number of mets./patient, mean/median(range)	1,5/1 (1–6)	1,8/1 (1–9)	0.073
Liver recurrence, LTP excluded	42/90 (47%)	30/62 (48%)	0.835
Follow-up time with imaging in months (standard deviation)	35,6 (18.9)	32,1 (13.3)	0.423
RFA (%) procedures	75/90 (83%)	51/62 (82%)	0.863
MWA (%) procedures	15/90 (17%)	11/62 (18%)	0.863
RFA (%) lesions	117/132 (88,6%)	94/111 (83.2%)	0.364
MVA (%) lesions	15/132 (11.3%)	17/111 (16.8%)	0.364
CEA, median (range)	3.4 (1–529)	3.9 (1.2–120)	0.897
ASA mean (SD)	2.42(0.52)	2.45 (0.50)	0.745

All procedures are reported and the table report gender, age and other parameters procedure-wise. Previous liver intervention includes resections and ablations, at least one and up to four previous interventions

In the inclusion period 12 patients had 2 procedures and one 4 procedures. 5 patients had treatment in both IVS and OR

### Local tumour progression

Local tumour progression outcomes are summarized in Table 2. Intraprocedural CT control was associated with significantly lower LTP rates on a per-lesion basis (5/111 [4.5%] in the CT group vs. 22/132 [16.6%] in the OR group) (*p* = 0.0035). The LTP rates were also lower per procedure in the CT group (5/62 [8.0%] vs. 20/90 [22.2%]) (*p* = 0.0255). Within the first year after ablation, LTP showed a nonsignificant trend towards lower rates in the CT group.

**Table 2:** Local tumour progression outcomes

	OR	CT lab	*p*-value
LTP per treated lesion	22/132 (16.6%)	5/111 (4,5%)	0.0035
LTP per treated lesion first year after ablation	14/132 (10,6%)	5/111 (4,5%)	0.0949
LTP per procedure	20/90 (22.2%)	5/62 (8.0%)	0.0255
LTP per treated lesion ≤ 10 mm	0/51 (0%)	1/50 (2%)	0.495
LTP per treated lesion > 10 mm or US-non-visualized lesion with fusion-based targeting	22/81 (27.2%)	4/61 (6.6%)	0.0018
LTP per treated lesion 11–20 mm	12/54 (22.2%)	2/42 (4.8%)	0.0196
LTP per treated lesion 21–30 mm	6/21 (28.5%)	0/11 (0%)	0.0711
LTP per treated lesion > 30 mm	1/1 (100%)	2/2 (100/%)	1
LTP per treated US-non-visualized lesion fusion-based targeting	3/5 (60%)	0/6 (0%)	0.0606

Group differences are significant by Fischer exact test except for lesion-wise LTP rate in the first post-ablation year, and for US-visualized CLM ≤ 10 mm in diameter

A procedure-based multivariate regression analysis with LTP as the dependent variable and group (CT or OR), RFA/MWA, size of the largest tumour, US visibility of lesion (fusion-based ablation), and number of treated lesions as covariates was significant (*p* < 0.001) and confirmed significant contribution from group (*p* = 0.010), tumour size (*p* = 0.002), and number of treated lesions (*p* = 0.005), whereas RFA/MWA and US visibility were not significant (*p* = 0.424 and 0.496, respectively). Lesion size strongly influenced outcomes. For US-visualized lesions ≤ 10 mm, LTP was rare in both groups (CT 2%, OR 0%; *p* = 0.495). For lesions > 10 mm or those requiring fusion-based targeting due to lack of US visibility, LTP was significantly lower in the CT group. Details of all lesions that developed LTP, including tumour size, number of lesions treated per procedure, visualization status, use of fusion-based targeting, and subsequent management, are summarized in Table 3.

**Table 3:** Details of lesions that developed LTP

	Number of CLM with LTP after ablation	Tumour size with later LTP, mm (median (range))	Number of treated CLM	Fusion-based punction of non-visualized CLM	LTP treated with ablation	LTP treated with resection	LTP not treated due to other synchronous metastases	LTP not treated due to LTP as dominant or only recurrence
CT lab, single CLM	1	35	1	0	0	1	0	0
CT lab, more than one CLM	4	13 (3–36)	2.5 (2–4)	0	1	1	1	1
OR, single CLM	11	21 (13–32)	1	0	2	1	7	1
OR, more than one CLM	10 *	13.5 (3–22)	3.5 (2–6)	3	3	3	4	1

Number of treated CLM in procedures where LTP later occurred, size of CLM with later LTP given as median and ranges and treatment of all LTP summarized. 3 fusion-based ablations of non-visualized tumour later had LTP.

*One ablation zone had 2 LTP treated with resection of LTP and a part of the ablation zone in one operation and later with ablation in a different location in the same zone. In total, LTP accounted for three cases of local untreatable tumour

### CT group

From a total of 62 procedures, re-ablations were performed after CT control in the same session in 32 procedures. In one of these procedures, three re-ablations were performed following three CT control scans and in another case, two re-ablations were performed after 2 CT control scans. In the remaining 30 procedures, a single re-ablation was performed after CT control, adding up to 35 re-ablations in total. One re-ablation was performed due to remnant tumour, one due to discovery of one additional metastasis on CT control and the remaining re-ablations were for margin extension. Confirmation CT after the last ablation was performed only after 4 of the 32 procedures. In 30 of 64 procedures, no need for re-ablation was detected. Five cases of fusion-based applicator placement for US/CEUS non-visualized lesions in six CRLM were noted in the CT group. 

Five LTP events occurred in the CT group, all within the first year after ablation. One LTP developed after a procedure without re-ablation, whereas four occurred after procedures where re-ablation had been performed based on CT assessment but without a subsequent confirmation CT. Four of the lesions had been treated with RFA and one with MWA. Three of the five LTPs occurred with synchronous intrahepatic recurrence elsewhere, and none followed fusion-based applicator placement. Management included successful repeat ablation in one case, surgical resection in two cases, and no further local therapy in two cases due to unresectable extensive intrahepatic disease. In one of these two cases, the LTP represented the dominant recurrence. One of the resected LTPs showed histology consistent with mass-forming cholangiocarcinoma. Pre-ablative and LTP-related characteristics are summarized in Table 3.

### OR group

From a total of 90 procedures, no remnant tumour was identified on CT-controls the day after the ablation procedure, and no re-interventions were performed. In this group, 5 cases of fusion-based applicator placement for US/CEUS non-visualized lesions were noted.

Twenty-two LTP events occurred following 20 procedures. Three LTPs occurred after fusion-based applicator placement. LTPs developed after treatment of both single (*n* = 11) and multiple CRLM (*n* = 10). One LTP occurred after MWA. Six LTPs occurred without concurrent intrahepatic recurrence, of which two had synchronous extrahepatic metastasis, whereas sixteen had concurrent intrahepatic recurrence. One ablation zone subsequently developed two temporally distinct LTP events requiring separate treatment. Five LTPs were treated with ablation, 2 with LTP as the only treatment indication. Three were successfully treated and 2 did not achieve local control (later untreated LTPs). Four LTPs underwent surgical resection, each combined with resection of additional liver metastases. In total, thirteen LTPs in 12 patients were not eligible for local therapy, primarily due to unresectable hepatic and/or extrahepatic disease, but two cases with LTP as the only recurrence were not treated due to insufficient liver reserve and general poor condition. Table 3 summarises the pre-ablative characteristics and subsequent LTP management.

## Complications

Complication rates did not differ significantly between groups. Complications occurred after 5 of 62 (8%) procedures in the CT group and 11 of 90 (12%) procedures in the OR group (*p* = 0.674). All events were Accordion grades 1 and 2. In the CT group, three grade 1 events and two grade 2 events were observed, consisting of four cases of unexpected post-procedural pain requiring analgesics and one self-limiting radial nerve paresis. In the OR group, six grade 1 events (pain) and five grade 2 events were recorded, including one bleeding episode, one pulmonary embolism, two cases of hepatic vein thrombosis, and one fever/infectious event.

## Survival and LTP-free survival

**Fig. 2:**
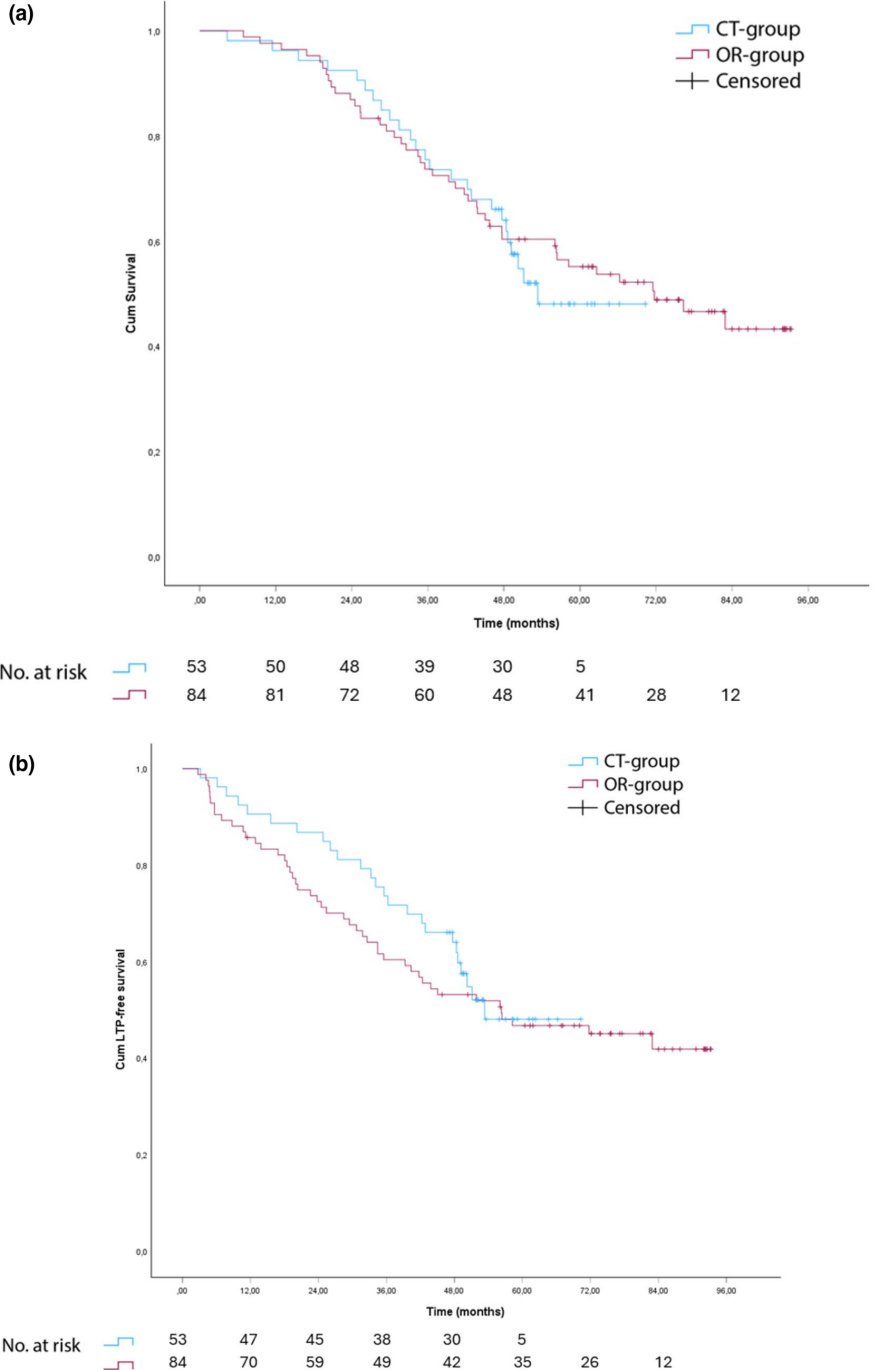
Kaplan–Meier plot of overall (**a**) and LTP-free (**b**) survival. CT group is blue, OR group is red. No significant difference between the two groups, *p* = 0.645 and *p* = 0.532, respectively

Overall survival and LTP-free survival did not differ between groups (*p* = 0.645 and *p* = 0.532, respectively). Kaplan–Meier survival curves are shown in Fig. 2.

## Discussion

This study demonstrated significantly reduced LTP rate with intraprocedural CT control after US-guided thermal ablation of CRLM. LTP was reduced on a per-lesion and per-procedure basis. These findings highlight the value of CT-based margin assessment also when ablation is performed under real-time US-guidance. No group difference regarding either overall or LTP-free survival was found, although an apparent separation of the curves is observed in the plot for LTP-free survival. Unequal observation time regarding survival is known to reduce the power of the log-rank test and in addition, the sample size might be too small to detect a difference.

The benefit of CT control is consistent with prior evidence showing that accurate assessment of ablative margins is critical for durable local control [5, 6]. Our study shows that margin assessment is more reliable with CT than with CEUS/fusion-based evaluation, despite no use of ablation confirmation software. In our cohort, inadequate margins remained the predominant explanation for LTP; notably, four of the five LTP events in the CT group occurred in procedures in which a final confirmation CT was not obtained after re-ablation. The motivation for not performing a confirmation scan was to limit the amount of CT contrast. In retrospect, we acknowledge that final CT-confirmation should be performed if possible, and we have changed our routine regarding this. Confirmation CT with reduced contrast dose is an option. The finding of LTP after re-ablation without final margin assessment reinforces the importance of end-of-procedure imaging and suggests that the benefit of CT control might have been even greater with routine confirmation scans and with the use of confirmation software.

Re-ablation was performed in more than half of the CT-group cases, which is more than the estimated reduction in LTP. All but two re-ablations were for margin extension, and visual evaluation of minimal ablation margin is reported to be inferior compared to use of confirmation software[12] Visual assessment may thus have contributed to this high re-ablation rate, and it is unclear if use of confirmation software would have reduced this rate.

Lesion size and visualization characteristics strongly influenced outcomes. LTP was uncommon in US-visualized lesions ≤ 10 mm and did not differ significantly between groups, supporting US-only ablation for small, well-visualized lesions. This is in accordance with previous findings [9]. In contrast, lesions > 10 mm or requiring fusion-based targeting had markedly higher LTP rates without CT control. Fusion-based targeting without CT verification resulted in LTP in three OR group lesions, whereas no LTP occurred in such lesions in the CT group. This suggests that CT is particularly valuable when US visualization is limited or when fusion accuracy may be compromised by tissue deformation.

LTP rate in the OR group is comparable to the MAVERRIC study, where stereotactic CT-guided MWA achieved survival comparable to local liver resection despite an LTP rate of 17% during the first year [3]. Stereotactic CT guidance in ablation has been increasingly adopted and comparative studies with US guidance are limited, but superior local control compared to US guidance has been reported in ablation of HCC [13]. The result of our study raises the possibility that inferior outcome with US guidance might be attributed more to inferior image control than imprecision in applicator placement, and this should be explored in the future studies.

LTP following CT-guided ablation has been reported by many, including studies of free-hand puncture technique, stereotactic guiding systems, and robotic-assisted needle placement. In a feasibility study on abdominal thermal ablations, the LTP rate at one year was 25.6% on a procedure basis, in a material where MWA on liver metastasis was the most frequent treatment. [14] The authors comment that the LTP rate is within the range reported in the literature but at the higher end. At the lower end, a 0% LTP rate over a follow-up period of 6 months was reported in an AmCORE-based study evaluating intra-tumoural contrast-tagging with CT hepatic angiography in CT-guided MWA of CRLM [15]. Ablation zone margins were calculated with confirmation software using rigid 3D image registration. In another recent study describing a purely CT-guided approach using MWA or RFA and a dedicated treatment planning system on liver malignancies 3 cm or less in size, residual tumour was reported in 9.3% of the cases [16]. A beneficial effect of intraprocedural CT-CT fusion for applicator and ablation assessment has been reported, with a reduction of lesion-wise LTP rate from 43% for a group treated without the use of image fusion to 15% with the use of image fusion [17]. Whether this reduction was attributed to improved applicator placement or improved margin assessment is unclear, but several studies have reported reduced LTP rates with the use of ablation confirmation software [5, 6, 18]. A recent review also concludes that ablation confirmation software for assessment of minimal ablative margins shows promising results [19]. The LTP rate in our cohort, also for our US-only procedures, is within what is reported in studies on CT-guided ablation, where there are variable results. In accordance with the reports of others, we find decreased LTP rate with improved margin assessment.

The majority of the LTP occurred in patients who also developed new liver metastases that were resected or ablated in the same session as the LTP or rendered the patient irresectable. In only one of the cases with concurrent metastasis, the LTP was the dominant recurrence and reason for no further treatment. However, isolated LTP occurred in six cases. Four received additional ablation or resection, and two were left untreated due to a small liver remnant and a frail patient, respectively, emphasizing the clinical relevance of avoiding preventable recurrences. Unlike other studies in which retreatment of LTP was frequently possible [3, 4], secondary ablation achieved durable control in only a minority of cases in our cohort, increasing the importance of obtaining adequate margins during the index procedure. Six patients underwent resection for LTP, five with simultaneous resection of additional liver recurrences. It could be questioned whether primary resection would have been preferable in these patients. However, in a parenchymal-sparing approach there is a trade-off between local tumour control and parenchymal loss. In a patient group where the majority will need repeated interventions, ablation as a first touch could be reasonable for ablatable tumours, especially where resection would imply a large parenchymal loss.

Superiority of MWA compared to RFA has been previously reported [8], and in our study, RFA predominates. In total, two LTPs occurred after 32 MWA (6.3%) and 20 LTPs after 211 RFA (9.5%). The difference is not significant, and the multivariate analysis did not identify the ablation method as a significant factor, but it cannot be ruled out that more frequent use of MWA could have improved the results. Currently, MWA is more frequently used in our institution than during the study period where inclusion ended in December 21. Although the cohort might not reflect contemporary practice, margin control remains essential, and adequate margins seem to be of greater importance than how margins are achieved.

This study has several limitations. Its retrospective, single-centre design limits generalizability, and treatment allocation was not randomized. Technically challenging cases were preferentially scheduled in the CT lab after this became available, which may have led to an underestimation of the true benefit of CT control. Although follow-up imaging was available for most patients, complete retrieval of externally performed scans was not possible due to data protective regulations, and late LTP in palliative patients may have gone undetected. The sample size in some subgroups, particularly fusion-targeted lesions and very large tumours, was limited, restricting interpretation. Finally, lesion-based analyses did not account for potential interactions between multiple metastases within individual patients.

In conclusion, intraprocedural CT control after US-guided ablation of CRLM was associated with a substantially lower LTP rate and should be considered standard when available. US-only ablation remains appropriate for clearly visualized lesions 10 mm or smaller, particularly where CT resources are limited. Patients with CRLM should be treated by a team with expertise in both resection and ablation.
